# Novel Secreted Peptides From *Rhizopus arrhizus* var. *delemar* With Immunomodulatory Effects That Enhance Fungal Pathogenesis

**DOI:** 10.3389/fmicb.2022.863133

**Published:** 2022-03-21

**Authors:** Sameh S. M. Soliman, Eman M. El-Labbad, Ameera Abu-Qiyas, Bahgat Fayed, Alshaimaa M. Hamoda, Ahmed M. Al-Rawi, Salam Dakalbab, Abdel-Nasser A. El-Shorbagi, Mawieh Hamad, Ashraf S. Ibrahim, Mohammad G. Mohammad

**Affiliations:** ^1^Research Institute for Medical and Health Sciences, University of Sharjah, Sharjah, United Arab Emirates; ^2^College of Pharmacy, University of Sharjah, Sharjah, United Arab Emirates; ^3^Pharmaceutical Chemistry Department, Faculty of Pharmacy, Ain Shams University, Cairo, Egypt; ^4^Pharmaceutical Sciences Department, College of Pharmacy, Gulf Medical University, Ajman, United Arab Emirates; ^5^Department of Medical Laboratory Sciences, Collage of Health Sciences, University of Sharjah, Sharjah, United Arab Emirates; ^6^Chemistry of Natural and Microbial Product Department, National Research Centre, Cairo, Egypt; ^7^College of Medicine, University of Sharjah, Sharjah, United Arab Emirates; ^8^Faculty of Pharmacy, Assiut University, Assiut, Egypt; ^9^Division of Infectious Diseases, The Lundquist Institute for Biomedical Innovation, Harbor-University of California at Los Angeles (UCLA) Medical Center, Torrance, CA, United States; ^10^David Geffen School of Medicine at UCLA, Los Angeles, CA, United States

**Keywords:** *Rhizopus arrhizus* var. *delemar*, secreted peptides, iron metabolism, immunomodulation, macrophages

## Abstract

Secreted fungal peptides are known to influence the interactions between the pathogen and host innate immunity. The aim of this study is to screen and evaluate secreted peptides from the fungus *Rhizopus arrhizus* var. *delemar* for their immunomodulatory activity. By using mass spectrometry and immuno-informatics analysis, we identified three secreted peptides CesT (S16), Colicin (S17), and Ca2+/calmodulin-dependent protein kinase/ligand (CAMK/CAMKL; S27). Culturing peripheral blood-derived monocytic macrophages (PBMMs) in the presence of S16 or S17 caused cell clumping, while culturing them with S27 resulted in the formation of spindle-shaped cells. S27-treated PBMMs showed cell cycle arrest at G0 phase and exhibited alternatively activated macrophage phenotype with pronounced reduction in scavenger receptors CD163 and CD206. Homology prediction indicated that IL-4/IL-13 is the immunomodulatory target of S27. Confirming this prediction, S27 initiated macrophage activation through phosphorylation of STAT-6; STAT-6 inhibition reversed the activity of S27 and reduced the formation of spindle-shaped PBMMs. Lastly, S27 treatment of PBMMs was associated with altered expression of key iron regulatory genes including hepcidin, ferroportin, transferrin receptor 1, and ferritin in a pattern consistent with increased cellular iron release; a condition known to enhance *Rhizopus* infection. Collectively, *R. arrhizus* var. *delemar* secretes peptides with immunomodulatory activities that support fungal pathogenesis. Targeting the IL-4/IL-13R/STAT-6 axis is a potential therapeutic approach to enhance the PBMM-mediated fungal phagocytosis. This represents a potential new approach to overcome lethal mucormycosis.

## Introduction

*Rhizopus* species are responsible for ~70% of all mucormycosis cases, a disease with high mortality rates in immunocompromised patients (Ibrahim et al., 2012). Recently, an outbreak of COVID-19 associated mucormycosis has been reported in India (Biswal et al., 2022). The mortality rate due to COVID-19 increased from 31 to 53% due to fungal infection including mucormycosis (Mehta and Pandey, 2020). Mucorales contamination of the hospital environment is among the significant risk factors for the outbreak (Biswal et al., 2022). *Rhizopus* spp. were the most common contaminates representing 67% from air-conditioning vents and masks, and 78% from the air (Biswal et al., 2022). Furthermore, successful management of mucormycosis requires early detection and aggressive treatment with antifungal drugs and often disfiguring surgery (Grant et al., 2006; Mohammadi et al., 2014). Hence, new strategies for the prevention and treatment of mucormycosis are urgently needed. A deeper understanding of the pathogenesis of *R. arrhizus* var. *delemar* (one of the most commonly isolated species from mucormycosis patients) should help in this endeavor.

Infections are associated with unique metabolic activities that modulate the host immune responses, which can be used as diagnostic/prognostic markers (e.g., C-reactive protein and procalcitonin; Ko et al., 2015). Similarly, the detection of a pathogen’s metabolic signatures during infection underlies several emerging approaches that can be used in disease diagnosis and treatment. The collection of peptides that are released into the extracellular space by a pathogen at any given time/condition (i.e., secretome) provides a unique avenue to study pathogen-specific metabolic signatures (Agrawal et al., 2010). Thus, immuno-informatics-based screening of secreted peptides along with experimental validation can help in identifying novel peptides of potential diagnostic and/or therapeutic value.

The robust secretory machinery of filamentous fungi has been widely studied (Conesa et al., 2001). However, little is known about the *R. arrhizus* var. *delemar* secretome, especially during conditions mimicking human infection. Filamentous microbes secrete macromolecules into the environment and alter the hosts they colonize (Kamoun, 2009). For example, fungi acquire nutrients and other elements that support their survival, growth, and pathogenesis by expressing and secreting degrading enzymes and other proteins and peptides that facilitate the decomposition and consumption of available organic matter (Girard et al., 2013). Proteins and peptides with no signal sequences are produced through exocytosis of coated vesicles, secretory lysosomes, microvesicles, and/or ATP-binding cassette transporters (Nombela et al., 2006). *Rhizopus* is a rich source of several enzymes including cellulases, hemicellulases, pectinases, tannases, phytase, amylases, lipases, and proteases (Ghosh and Ray, 2011). However, immunomodulatory peptides and proteins secreted by *R. arrhizus* var. *delemar* during mucormycosis are poorly investigated (Rabouille, 2017). Herein, we investigated the *R. arrhizus* var. *delemar* secretome when interacting with alveolar epithelial cells and under simulated conditions of infection. Short peptides with predicted immunomodulatory activities on PBMMs were further characterized.

## Materials and Methods

### Fungal Cultures

*R. arrhizus* var. *delemar*-99880, isolated from the brain of a patient with mucormycosis, was obtained from the Fungus Testing Laboratory, University of Texas Health Science Center, San Antonio, TX, United States. The fungus was grown on two different culture media to simulate initiation and hematogenous dissemination of infection. For the initiation of infection condition, 12-well plates were seeded with 4 × 10^3^ alveolar epithelial cells (A549) per 100 μl F12K-medium and incubated at 37°C for 24 h in 5% CO_2_ until confluency. The wells were then washed and incubated for 3 days with fungal spores (5 × 10^6^) suspended in fresh F12K media. A549 cells suspended in F12K medium containing no fungal cells served as a control. For the hematogenous dissemination-like condition, fungal spores were grown submerged in potato dextrose (PD) broth at 37°C without shaking for 24 h to create a hypoxia-like culture condition.

### Prediction Software

*Rhizopus* fungal genome was checked for the presence of signal peptide sequences using SignalP 3.0 software available online and by employing hidden Markov models (HMM) trained on eukaryotes (Nielsen and Krogh, 1998; Dyrløv Bendtsen et al., 2004). Peptides’ properties were calculated using PepCalc.com https://pepcalc.com/ (Lear and Cobb, 2016). Prediction of linear cationic antimicrobial peptides (AMP; Vishnepolsky and Pirtskhalava, 2014) was performed using DBAASP at https://dbaasp.org/prediction. The sub-cellular localization of the identified peptides was predicted by DeepLoc-1.0 software http://www.cbs.dtu.dk/services/DeepLoc/ (Almagro Armenteros et al., 2017). To detect the presence of linear antigenic epitope motifs from identified peptides, Support Vector Machine-Tri-peptide (SVMTriP), an online prediction tool was employed http://sysbio.unl.edu/SVMTriP/prediction.php (Yao et al., 2012). The overall antigenicity and predicted antigenic probability of the epitopes predicted by SVMTrip was validated by ANTIGENpro http://scratch.proteomics.ics.uci.edu/ (Magnan et al., 2010). Comparative amino acid sequence analysis between peptides and IL-13 or IL-4 was performed using default parameters of “align sequence” protocol using IL-13 (Accession# P35225) and IL-4 (Accession# AAX36848). Sequence alignment of the tested proteins was performed using ClustalOmega and MuscleWS. Phylogeny relationship of the tested peptides was conducted by neighbor-joining tree using BLOSUM62. Percent identity was measured using Pairwise alignment. Jalview 2.11.1.4 software was used for visualization. Phosphorylation domain was predicted using NetPhos-3.1 available at https://services.healthtech.dtu.dk/service.php?NetPhos-3.1.

### Protein Identification and Peptides Synthesis

Supernatants collected from cultures grown under initiation of infection or hematogenous dissemination conditions were purified from fungal hyphae by centrifugation at 1.00 xg for 5 min followed by filtration using membrane filters with a molecular weight cut off (MWCO) value of 0.2 kDa (Cheung et al., 2015). Concentrated supernatants were resolved by in-gel protein separation using SDS-polyacrylamide gel electrophoresis. In-gel protein bands were digested overnight at 37°C using Trypsin Gold, Mass Spectrometry Grade (Promega, Fitchburg, WI, United States). Digested proteins were cleaned using resin columns (Thermo Scientific™ Pierce™ Detergent Removal Spin Columns, Cat # 10499764) and eluted in 0.1% formic acid solution followed by chromatography–tandem mass spectrometry (LC–MS/MS) analysis (Liu et al., 2015).

The peptides with predicted promising pathogenic activities were synthesized (GenScript, NJ, United States). The purity of the peptides was tested and validated with HPLC. The lyophilized peptides were suspended in PBS or DMSO according to the manufacturer’s instructions at 1 mg/100 μl stock and stored at −80°C.

### Screening for the Antimicrobial Activity

To screen for the antimicrobial activity, disc diffusion assay was employed according to the Clinical and Laboratory Standards Institute (CLSI) guidelines (CLSI, 2015; Zhang et al., 2009) and Soliman et al. (2017). Briefly, 6 mm sterile filter paper discs (Whatman, no. 3, fisher scientific) were soaked with 10 μl of concentrated culture extract and then placed on the surface of Laurel broth (LB) agar plates streaked with 100 μl of *E. coli* bacterial suspension containing 1.5 × 10^8^ CFU/ml. The plates were incubated in the dark at 37°C for 24 h. The clear zone (zone of inhibition) surrounding the filter discs were measured in millimeter; all tests were performed in triplicate.

### Mammalian Cell Damage Assay

A549 cell damage was quantified using ^51^Cr release assay (Ibrahim et al., 2008). Briefly, cells grown in 96-well tissue culture plates containing detachable wells were incubated with 1 μl ^51^Cr/well Na_2_^51^CrO_4_ (ICN) in F12K-medium for 16 h. The unincorporated ^51^Cr was aspirated, and the wells were washed twice with pre-warmed HBSS. The cells were treated with the compound suspended in F12K-medium supplemented with glutamine and incubated at 37°C in 5% CO_2_ incubator. Spontaneous ^51^Cr release was determined by incubating the A549 cells only in culture medium supplemented with glutamine. At different time points, the medium was aspirated from each well and transferred to glass tubes, and the wells were manually detached and placed into another set of tubes. The amount of ^51^Cr in the aspirate and the detached well was determined by gamma counting. The total amount of ^51^Cr incorporated by the cells in each well was calculated as the sum of radioactive counts per min of the aspirated medium and radioactive counts of the corresponding detached wells. After data were corrected for variations in the amount of tracer incorporated in each well, the percentage of specific cell release of ^51^Cr was calculated as follows: [(experimental release) − (spontaneous release)]/ [1 – (spontaneous release)]. Each experimental condition was tested in triplicate (at a minimum) and the experiment was repeated three times.

### Peripheral Blood-Derived Monocytic Macrophages Collection

Blood samples from healthy donors were collected after signing an informed consent forms authorized by the University of Sharjah ethics committee (ethical approval # REC-19-07-19-01). Blood was collected in EDTA tubes and pooled into 50 ml falcon tube. To enrich for PBMMs, 12.5 ml pooled blood was over-layered onto 10 ml Histopaque-1,077 (Sigma-Aldrich, St. Louis, MO) followed by centrifugation at RCF 400 for 25 min at room temperature with brakes off. Peripheral blood mononuclear cells (PBMCs) in the interface were then collected and washed one time with phosphate-buffered saline (PBS). Viable cells were counted using trypan blue vital dye. To measure cell proliferation, cells were seeded into 96-well tissue culture plates at a density of 5 × 10^3^ cells per 200 μl of RPMI-1640 media supplemented with 10% fetal bovine serum (FBS; Sigma) and 1% penicillin/streptomycin (Sigma-Aldrich, St. Louis, MO). For cell cycle analysis, cells were seeded into 24-well tissue culture plates at a density of 2×10^5^ cells/ml RPMI-1640 media supplemented as noted above. At least four wells were employed for each treatment in every experiment.

For Western blotting and flow cytometry, cells were seeded into 6-well tissue culture plates at a density of 2.5×10^6^ cells/ml RPMI-1640 media supplemented as previously mentioned. At least three wells were employed per treatment per experiment. Plates were incubated for 24 h at 37°C and 5% CO_2_. Floating non-monocytic cells were removed by gentle aspiration of the supernatants followed by washing with pre-warmed PBS. RPMI-1640 alone (employed as control) or containing the peptides was then added to the attached PBMMs.

### XTT Proliferation Assay

XTT proliferation assay (Intron Biotechnology, South Korea) was used to detect the mitochondrial activity as a proxy of PBMMs proliferation in response to peptides S16, S17, and S27 (Kairo et al., 1999). XTT was applied to cultured PBMMs according to the manufacturer’s instructions. In brief, PBMMs were incubated with the peptides at a final concentration of 10 and 100 ng/ml. Lipopolysaccharide (LPS) was employed as a positive control at a final concentration of 100 ng/ml. PBMMs were incubated with the peptides or LPS for 24 h. At the end of incubation, XTT was added for 4 h followed by measuring the absorbance at 450 nm using a standard microplate spectrophotometer (BioTek, Vermont, United States).

### Cell Cycle Analysis

PBMMs were incubated with the peptides at a final concentration of 10 and 100 ng/ml in a final volume of 500 μl. LPS, which was used at a final concentration of 100 ng/ml, served as a positive control, non-treated and vehicle-treated cells were employed as negative controls. After 24 h of treatment, the supernatants were removed, and the cells were fixed for 30 min on ice by adding 1 ml of 70% ethanol dropwise to prevent cell clumping. The cells were then washed twice, and the ethanol was replaced with 500 μl PBS containing propidium Iodide (PI)/RNAse staining buffer (BD Biosciences) to label the DNA of treated PBMMs. Labeled cells were run through AreaIII flow cytometer (Beckton Dickinson, United States); gating strategy and flow cytometry data were analyzed using the FlowJo software (BD, United States; Supplementary Figures 1, 2).

### Morphological Observation of Macrophages

To test for morphological changes in peptide-treated macrophages as a proxy of their activation, PBMMs in RPMI-1640 media were incubated with the S27 peptide (1, 10, 50 ng/ml) ± STAT-6 antagonist (AS1517499, Sigma-Aldrich, Netherlands) at 1 μM (Kim et al., 2018). Interleukin 4 (IL-4) treatment was employed at 15 ng/ml as a positive control. A mixture of IL-4 (15 ng/ml) and STAT-6 antagonist (1 μM) was used as an additional control for the inhibition of IL-4-mediated macrophage polarization. Untreated cells were employed as a negative control, and each treatment was done in triplicate. At day 8 post culture with the respective treatment, the morphology of macrophages was examined by light microscopy (10x magnification) using IX73 inverted Olympus microscope (Olympus, Japan). Images were taken from random fields for each treatment utilizing the Olympus cellSens Entry software. Images were then analyzed, and spindle-shaped macrophages were counted using ImageJ software. Counts were normalized based on count of spindle-shaped cells per unit area.

### Flow Cytometry Analysis

Primary cultures of 24-h-incubated PBMMs were treated with S27 peptide at 1, 10, and 50 ng/ml. LPS and IL-4 were employed as positive controls at 100 and 15 ng/ml, respectively, and each treatment was done in triplicate. PBMMs were incubated with the respective peptide for 8 days. Macrophages were then harvested by gentle scraping of culture plates with 1 ml of ice-cold PBS; this was repeated several times to ensure that all cells were harvested. Cells were then transferred to 15 ml falcon tubes followed by centrifugation at 0.450 × *g* for 10 min at 4°C. Supernatants were discarded and pellets were re-suspended in freshly prepared 500 μl staining washing buffer (SWB) containing 1% FBS and 1 μM EDTA in PBS. Cell counts were determined using trypan blue vital stain using Neubauer improved hemocytometer. Macrophages were incubated for 30 min on ice, in the dark in SWB with a fluorophore-conjugated monoclonal antibody master mix containing CD14, CD206, CD163, HLA-DR (BD, United States), CD16, and CD86 (R&D, United States). Cells were fixed using the fixation/permeabilization solution (Cytofix/Cytoperm kit, BD Biosciences) for 20 min on ice in the dark. Dot plots of forward and side scatter were used for initial gating of cells followed by single-cell population gating. Then, CD14 and CD16 cell populations were selected followed by gating for each specific marker versus the general macrophage population (either CD14 + CD16− or CD14 − CD16+; Supplementary Figures 1, 2). Samples were then washed once, and pellets were re-suspended in 250 μl SWB. Sample analysis was performed using FACS Area III; acquired data were analyzed using the FlowJo v10 analysis software (BD, United States).

### Western Blot Analysis

The ability of S27 peptide to initiate the phosphorylation of STAT-6 signaling pathway was determined using Western blot analysis. PBMMs were prepared as described above. Cells were incubated with IL-4 (15 ng/ml) or S27 (100 ng/ml) for 15 min, 1, and 4 h. Cells were also incubated with LPS (100 ng/ml) for 15 min as a negative control of STAT-6 phosphorylation. To further characterize any functional changes precipitated by the S27 peptide in PBMMs, the expression of key proteins involved in iron homeostasis was assessed in cells cultured with the S27 peptide (at 10 ng/ml) in the presence or absence of STAT6 antagonist (at 1 μM). At day 8 of the treatment, cells were harvested and lysed using RIPA buffer. Protein concentration was estimated using Pierce™ BCA protein assay kit (BioRad) following the manufacturer’s manual. Samples were denatured by adding Laemmli buffer containing 2-mercaptoethanol at 95°C for 5 min, then the samples were loaded on 8.5% SDS–polyacrylamide gel electrophoresis and transferred to nitrocellulose membrane. Subsequently, the membrane was blocked by a blocking buffer (Tris-buffered saline buffer containing 0.1% Tween-20 and 5% non-fat dry milk) for 30 min. Membranes were then washed and incubated with primary antibodies against Phospho-STAT-6 (Tyr641; #9361, Cell Signaling), STAT-6 (D3H4; #5397, Cell Signaling), ferroprotein (FPN; 1:1000, NBP1-21502, Novus Biologicals), hepcidin-25 (HEP; 1:250, ab30760, Abcam), transferrin Receptor (TfR-1; 1:1000, ab84036, Abcam), ferritin (Ferr; 1:1000, ab75973, Abcam), or heamoxygenase-1 (HO-1; 1:1000, MA1-112, Invitrogen). A monoclonal antibody against β-actin (#4970, Cell Signaling) was used as a loading control. Cells were washed with PBS and horseradish peroxidase-conjugated secondary antibodies (#7074S, Cell signaling) were added and incubated for 1 h. Protein bands were detected by an enhanced chemiluminescence (ECL) detection kit (#170–5,060, Bio-Rad) and visualized by Bio-Rad ChemiDoc gel imaging system (Eldohaji et al., 2021). Band density was measured using ImageJ software and normalized to β-actin.

### Statistical Analysis

All performed experiments were analyzed using GraphPad Prism software (version 9.0.0; GraphPad Software, San Diego, California United States) and results were expressed as the mean ± standard error of the mean (SEM). Quantitative comparisons between groups in all data of proliferation assay, flow cytometric analysis, and densitometry of western blots were analyzed by one-way analysis of variance (ANOVA). Cell cycle analysis and morphological counts were analyzed by two-way ANOVA. *p*-value ≤0.05 represented significant statistical difference.

## Results

### *In silico R. arrhizus* var. *delemar* Genome Mining Revealed ORFs With Predicted Signal Peptides

To investigate the secretion of pathogenic peptides by *R. arrhizus* var. *delemar*, the fungal genome was screened for the presence of gene sequences containing signal peptides (SP) in the *R. arrhizus* var. *delemar* genome. As shown in Table 1, 10 predicted protein sequences (S1-S10) with SP were identified including hypothetical proteins with homology to phospholipase DDHD2, heat shock protein DnaJ, proteophosphoglycan5, ribosomal protein, LysM domain-containing protein, exonuclease SbcC, membrane protein histidine kinase (HK), transposon protein along with two *R. arrhizus* var. *delemar*-specific hypothetical proteins; one of which showed homology to CotH3, a cell surface protein required for full virulence (Table 1).

**Table 1:** Predicted protein sequences with signal peptides.

Sequence #	Accession number	SP sequence	Highest homology	
			Homology	Identity
S1	RO3G_10006	MEMILFSFFFFLFFSCTIIAKYMNSVIDLPN EILLAIFGLLTPVDQFNCQLVC QAWLMSSRQIYYEKVKT	Phospholipase DDHD2	17%
S2	RO3G_09031	MRSSKRLLFAFVLPFFINHQVTAFEKDAKF YLEEGNQYLSSGKFNDAILSYD TAIQQDPSDYLSYYKRAT	Heat shock protein DnaJ	92%
S3	RO3G_14304	MHLSSMNKIYLLLLIVAALLGFSAEAGLLS YVICQTGCNTLDATCYAASGLT FGTVTTGAGAPAVALAYN	Proteophosphoglycan5	73%
S4	RO3G_16353	MFHQMRYACSHLLSGIILLDTLTGKTVIINS VEVFGRRSSLDAHRKSFRVKKE AEVSTSLPPEITRYRNV	Hypothetical	92%
S5	RO3G_02516	MKLAVYLTLLFAAVIMMATAAPHNKSCHR LKDPHANAVCKAYCGKAGYKL GECGLQGICICKKTKISTKV	CotH3	100%
S6	RO3G_12370	MINFKAYRAILLLVILSLDCALSDELLPRRSL PVKHRWMKLPRRKPKKSLFNR FVPDFADPHRCEPKQLA	Putative 40S ribosomal protein S16	77%
S7	RO3G_02853	MKFAIAATALAFVSAASALSTNCGEEYIAKE GDTCSSIAADKKISIQSLYRLNVD NLNIGDCTALEAGKP	Secreted LysM domain-containing protein	35%
S8	RO3G_11226	MDNRYPYTNSRDQPHPPSTSPDKTHWQPTQ LYPPAMLHTPPFPYGHFTHFPRV PHPSPDVSYPHHTRQDP	Exonuclease SbcC	-
S9	RO3G_08085	MPTIDRAVIWANEFNDLQAPDLKTRQQAVL KAKMEASESDDSLLIMYTDFLESV LKFYKTLNVNDTKDNA	Membrane protein histidine kinase	-
S10	RO3G_00492	MSNLQDLLKSDSEDELTRYYDGNRKRQKCE ENEEDDIATFLAIDIITEEFSSWQE KRRGGSVVGRRVIPR	Transposon protein	69%

*The highlighted sequences represent the signal peptide*.

### *R. arrhizus* var. *delemar* Secretes Variable Peptides When Grown Under Conditions Simulating the Initiation of Infection vs. Hematogenous Dissemination

**Figure 1:**
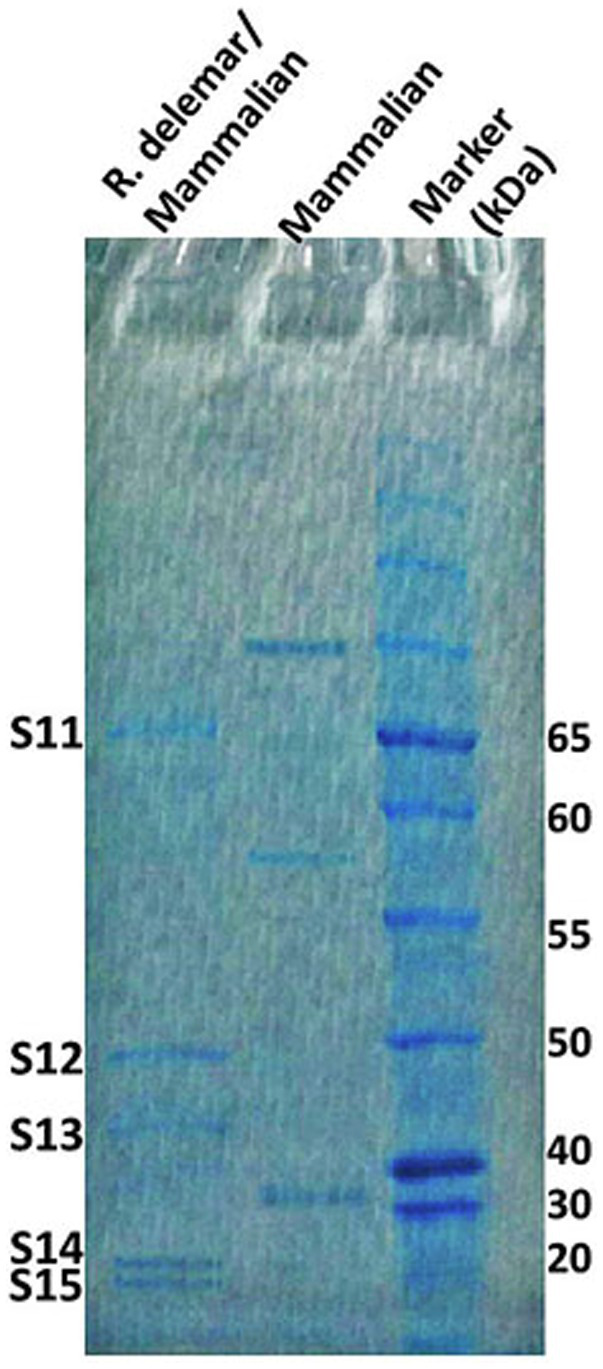
*R. arrhizus* var. *delemar* secretome analysis in relation to initiation of infection. SDS gel electrophoresis of culture supernatants collected from mammalian cell cultures and co-cultures of *R. arrhizus* var. *delemar* and lung epithelial cells.

**Figure 2:**
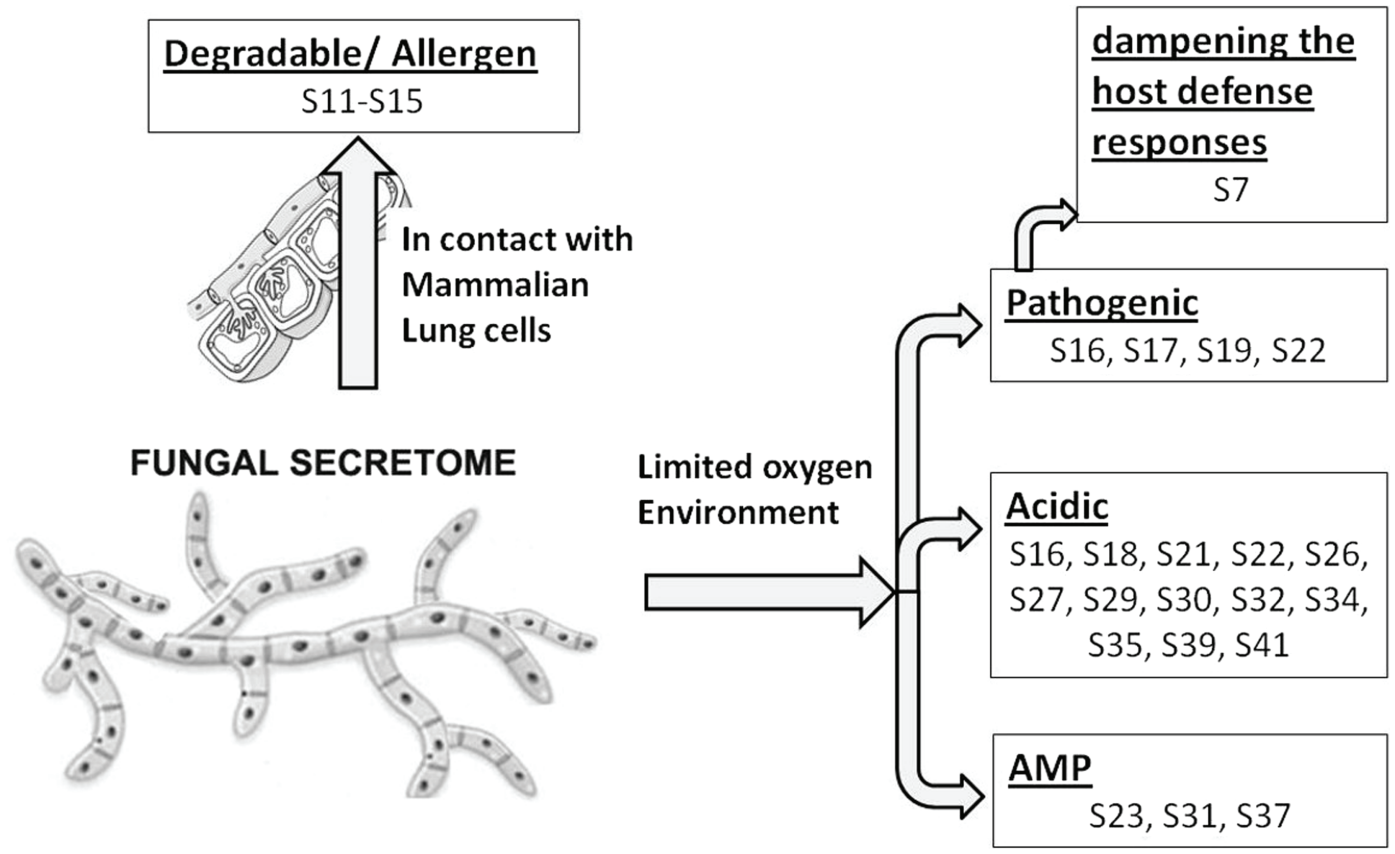
Model representing *R. arrhizus* var. *delemar* secretome analysis in response to growth at two different environmental conditions (initiation- and dissemination-like conditions).

In response to A549 cells, *R. arrhizus* var. *delemar* secreted four major and one minor protein bands (Figure 1). These protein bands were excised, trypsinized, sequenced, and identified (S11-S15) as glucoamylase-1, enolase, lipase, and two uncharacterized proteins with one of which being homologous to chitin deacetylase (Table 2). In contrast, growing *R. arrhizus* var. *delemar* in PD broth under limited oxygen supply to mimic simulation of disseminated infection, revealed the presence of several peptides (S16–S41; Table 3). Most of the identified peptides were predicted to be processed from gene products annotated as hypothetical proteins but showed homology to known secretory proteins and/or toxins including CesT, Colicin, T9SS, Heat-labile enterotoxin IIA, and 5′-3′ exoribonuclease. Additionally, some peptides were computationally predicted as acidic, pathogenic, immunogenic, and antimicrobial (AMP) peptides, such as type I secretion system and exoribonuclease. All identified peptides and proteins in relation to their predicted virulence activities are summarized in Figure 2.

**Table 2:** Protein sequences identified by LC–MS/MS in the supernatant of *R. arrhizus* var. *delemar* fungus growing in contact with lung epithelial cells compared to controls, the fungus and mammalian cells alone.

Protein band	MW [kDa]	Peptides/AA	E value	Score/Coverage	Accession number	Highest homologue	Presence or absence (Score/coverage)	
							Other Mucorales	Aspergillus
S11	65.0	25/604	1.151E^10^	316.29 /46.36%	RO3G_00082	Glucoamylase-1	Yes	291/40%
S12	47.0	28/437	2.651E^9^	312.13 /67.05%	RO3G_11989	Enolase	Yes	No
S13	42.1	20/ 392	4.715E^10^	504.30 /60.71%	RO3G_00087	Lipase	Yes	No
S14	21.2	22/ 189	8.031E^9^	318.96 /70.90%	RO3G_01608	Uncharacterized similar to Chitin deacetylase	Yes	No
S15	20.6	18/ 181	5.518E^9^	235.11 /59.12%	RO3G_11298	Uncharacterized	No	221/58%

**Table 3:** Identification of peptides sequences by LC–MS/MS in the supernatant of *R. arrhizus* var. *delemar* fungus growing at dissemination-like condition compared to control culture media without the fungus.

Sequence #	Detected AA sequence	Predicted Accession number	MW [kDa]	Calc. pI	Identity	Homology	Acidic/ Basic	AMP
S16	FADNSIIIPCRALDSQMEVIR	RO3G_03117	2.4	4.17	Hypothetical	CesT (type III secretion system)	Acidic	NO
S17	ESHLVMTESLIRNMKQR	RO3G_06582	2.01	10.1	Hypothetical	Colicin, a voltage gated bacterial toxin	Basic	NO
S18	IRFDDDLLENAQNSLETLVRVGNHIAVQV	RO3G_11316	3.3	4.07	Hypothetical	-	Acidic	NO
S19	ERQQIK	RO3G_07458	0.8	10	Hypothetical	T9SS (type IX secretion system)	Basic	NO
S20	AVPVGGIAYYLSAPQGLMDAVFHPVHTLVYASLTIITCAYLSK	RO3G_01407	4.5	7.2	SecY	SecY protein	Basic	No
S21	NLDVGATLFIGNLDPEVDEKILYDTFSAFGLIVNTPR	RO3G_10200	4.1	3.5	Hypothetical	Splicing factor 3B	Acidic	NO
S22	LVDSCAELNK	RO3G_10210	1.1	3.9	Hypothetical	Heat-labile enterotoxin IIA	Acidic	Yes
S23	KLSEIPR	RO3G_06209	0.9	9.9	Hypothetical	Type I secretion system	Basic	Yes
S24	GTQIYSPEK	RO3G_02202	1	6.8	Hypothetical	Brl domain-containing protein	Neutral	NO
S25	LKSSLPR	RO3G_11393	0.8	11.4	Cyclin	Secretion protein HlyD	Basic	NO
S26	AVMWLSPIYPVDFNYQIKMVSLAEYQNTVCK	RO3G_02650	3.6	6.2	Hypothetical	UDP-Glycosyltransferase	Acidic	NO
S27	LLQLSEPPVSELDQLTYNNTMFTNNKITTSHTATPREFR	RO3G_04287	4.5	5.4	Hypothetical	CAMK/CAMKL protein kinase	Acidic	NO
S28	VTHVNEQMAPFHILWPHFHQYRFQNVMTDLFLRNHHL	RO3G_01169	4.6	8.12	Hypothetical	Not identified	Basic	NO
S29	EAEELAEDLGIKIFTADIIYHLFDRFQEHFAAIAEQK	RO3G_03428	4.3	4	Hypothetical	Putative Eukaryotic translation initiation factor 5B (Rhizopus microsporus)	Acidic	NO
S30	NDLVIRLRFDWLVDYEQQR	RO3G_05799	2.5	4.3	Hypothetical	type II/IV secretion system protein	Acidic	NO
S31	EGAISTLSLLWKKCMPMMGGYMTK	RO3G_06774	2.7	9.6	Hypothetical	5′-3′ exoribonuclease-Lichtheimia corymbifera-91% identity	Basic	Yes
S32	EGIKNGDIQDERPWDTKDFDLCR	RO3G_13952	2.8	4	Hypothetical	CST complex	Acidic	NO
S33	LKSSLPR	RO3G_11393	0.84	11.4	Not Identified	Neurobeachin-like protein 2	Basic	NO
S34	IFDSLNYVENMRLGIDAVK	RO3G_06931	2.2	4.2	pyruvate carboxylase	pyruvate carboxylase	Acidic	NO
S35	ESEEESEEERQEELSDmEGYDAPTDLTEFGVmSSKK	RO3G_14100	4.2	3.5	Hypothetical	RNA-binding ATPase activator esf2	Acidic	NO
S36	NGIALAELLLFDQRIVANLVRFMK	RO3G_10507	2.8	9.7	Hypothetical	Dedicator of cytokinesis protein	Basic	NO
S37	KTRSTSYFNVLFSGFALLSDGYQSGVISFVNLFLGK	RO3G_07416	3.9	9.9	Hypothetical	MFS transporter	Basic	Yes
S38	SLAYETQMGIK	RO3G_02956	1.3	6.6	Hypothetical	TonB-dependent receptor	Neutral	NO
S39	AVELSQFFVSSVLSRCCSAEQSDDFWPVDVLLYLMSR	RO3G_15074	4.3	3.7	Hypothetical	Hypothetical	Acidic	NO
S40	FLLLPPQATVKQRTIIIR	RO3G_14307	2.2	12.1	Hypothetical	regulator of G protein signaling	Basic	NO
S41	ELSRVSRMSSVHAGALESGNAASDEFNLDEFLNGLR	RO3G_15968	3.9	4.3	Hypothetical	69% identity to ABC transporter G family member 14	Acidic	NO

*AMP, Antimicrobial peptide*.

**Figure 3:**
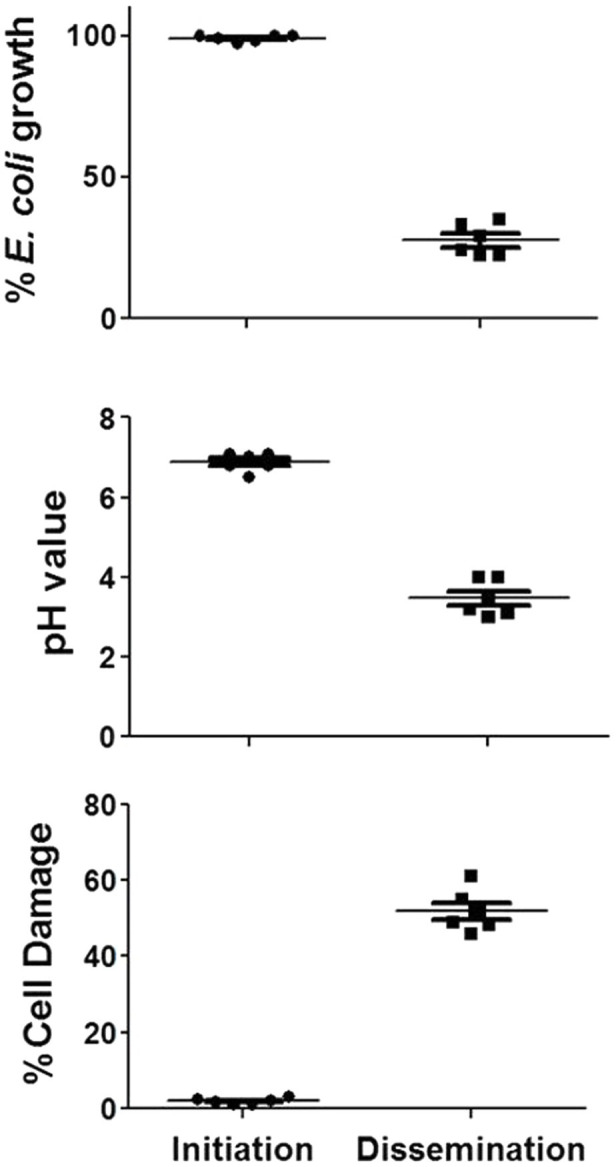
Activity of *R. arrhizus* var. *delemar* cultures supernatants. Mammalian cell damage, pH values and anti-*E. coli* activities of concentrated *Rhizopus* PD (dissemination-like) versus F12K (initiation-like) cultures. All values are relative to control culture media without the fungus.

Based on these data, the activities of concentrated PD cell-free supernatants from *R. arrhizus* var. *delemar* grown under initiation or dissemination-like infection conditions on host cell or bacterial cell growth were assessed compared to control culture media without the fungus. While supernatants collected from cultures that simulated the initiation of infection did not cause A549 cell damage, extracts from supernatants collected from cultures that simulated dissemination of infection showed considerable (>50%) mammalian cell death (Figure 3). Furthermore, extracts from dissemination-like condition cultures caused pH reduction to 3.4 and anti-bacterial activity (>70% growth inhibition) against *E. coli* (Figure 3).

### Secreted Peptides From a Hematogenously Disseminated-Like Condition Were Identified by Filtering the Secretory Features Using Computational Sub-Cellular Localization

**Figure 4:**
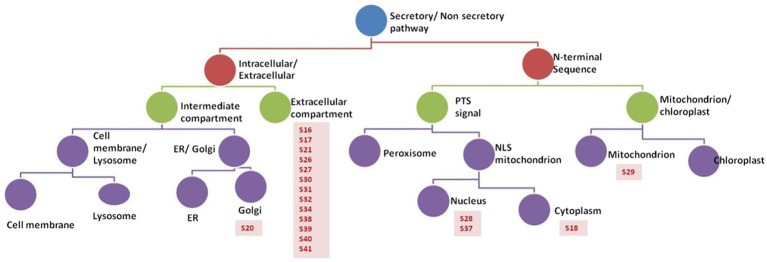
Analysis of peptides secreted by *R. arrhizus* var. *delemar*. Prediction analysis of the sub-cellular localization of the *R. arrhizus* var. *delemar* small peptides was carried out using DeepLoc1.0 (Almagro Armenteros et al., 2017; http://www.cbs.dtu.dk/services/DeepLoc/instructions.php).

Prediction analysis of the sub-cellular localization of identified peptides (S16-S41) was performed using DeepLoc-1.0 software, which utilizes deep recurrent neural networks for protein sub-cellular localization with convolution motif detectors, selective attention on sequence regions important for sub-cellular localization, and hierarchical sorting among other features (Almagro Armenteros et al., 2017). Since the cutoff number of the peptide sequence required by Deeploac-1.0 is 10 amino acids, Deeploac-1.0 was used only to predict the sub-cellular localization of 19 small peptides. Only one peptide (S20) is predicted to be a membrane bound peptide localized in Golgi apparatus, while the other 18 peptides were predicted to be soluble peptides (Figure 4). Out of the 18 peptides, 13 peptides (S16, S17, S21, S26, S27, S30, S31, S32, S34, S38, S39, S40, and S41) are predicted to be extracellular, two (S28, S37) are predicted to localize in the nucleus, S18 is predicted to be localized in the cytoplasm, and S29 is predicted to be a mitochondrion (Table 3).

### Three Extracellular Peptides Secreted in Hypoxic Conditions Were Predicted to Be Antigenic

To predict the antigenicity of the identified secreted peptides (S16, S17, S21, S26, S27, S30, S31, S32, S34, S38, S39, and S41), we subjected the peptides to *in silico* screening to identify antigenic epitopes that can be preferentially recognized by the B-cell antibody repertoire (Getzoff et al., 1988). The overall antigenic probability of epitopes was predicted by SVMTriP and further calculated using ANTIGENpro. SVMTriP predicts the linear antigenic epitope utilizing Support Vector Machine learning approach (SVM) combining the Tri-peptide similarity and propensity scores using non-redundant B-cell linear epitopes extracted from IEDB epitope database (Yao et al., 2012). ANTIGENpro predicts the whole protein/peptide antigenicity based on human immunoglobulin reactivity data obtained from protein microarray analyses using antigens from five pathogens that elicit a strong antibody response in protected individuals but not in unprotected individuals (Magnan et al., 2010). Our data showed that ANTIGENpro prediction algorithm verified the antigenicity of 10 epitopes with a probability prediction range of 0.129400–0.586166 (Table 4). Three predicted antigenic epitopes with 10 amino acid sequences derived from peptide S16 (PCRALDSQME), peptide S17 (TESLIRNMKQ), and peptide S27 (VSELDQLTYN) showed promising antigenic probability of 0.528628, 0.586166, and 0.567569, respectively. SVMTriP also predicted the underlined 10 amino acids (MGQTNDGAYRDPTDNN) as possible linear epitopes derived from CotH3 with antigenic probability value of 0.558507, which is comparable to epitopes derived from S16, S17, and S27. This CotH3 epitope was previously described as an antigenic surface-exposed peptide from *R. arrhizus* var. *delemar* 99–880 (Gebremariam et al., 2014). Hence, the confidence of the results was obtained by the use of SVMTriP and ANTIGENpro prediction analyses and in comparison to CotH3.

**Table 4:** *In silico* prediction of linear antigenic epitopes and the probability of antigenicity originated from peptides showing predictive extracellular localization.

SVMTriP predicted linear antigenic epitope^a^	ANTIGENpro predicted probability of antigenicity of epitope^b^	Peptide of origin
PCRALDSQME	0.528628	S16
TESLIRNMKQ	0.586166	S17
GATLFIGNLD	0.129400	S21
TFSAFGLIVNT	0.167900	S21
LVDSCAELNK^*^	0.421030	S22
QIKMVSLAEY	0.358044	S26
VSELDQLTYN	0.567569	S27
ENMRLGIDAV	0.406612	S34
NGIALAELLLFDQRIVANLV	0.286975	S38
TVKQRTIIIR	0.442321	S40
NDGAYRDPTD^**^	0.558507	CotH3

a *http://sysbio.unl.edu/SVMTriP/prediction.php*.

b *http://scratch.proteomics.ics.uci.edu/*.

* *LVDSCAELNK is the full sequence of Secreted protein S22*.

** *NDGAYRDPTD is SVMTriP Predicted linear antigenic epitope originated from CotH3 (MGQTNDGAYRDPTDNN), a reported antigenic and surface-exposed protein from R. arrhizus var. delemar 99–880*.

### *R. arrhizus* var. *delemar* Peptide S27 Induces Non-classical M2-Like Polarization in Macrophages

**Figure 5:**
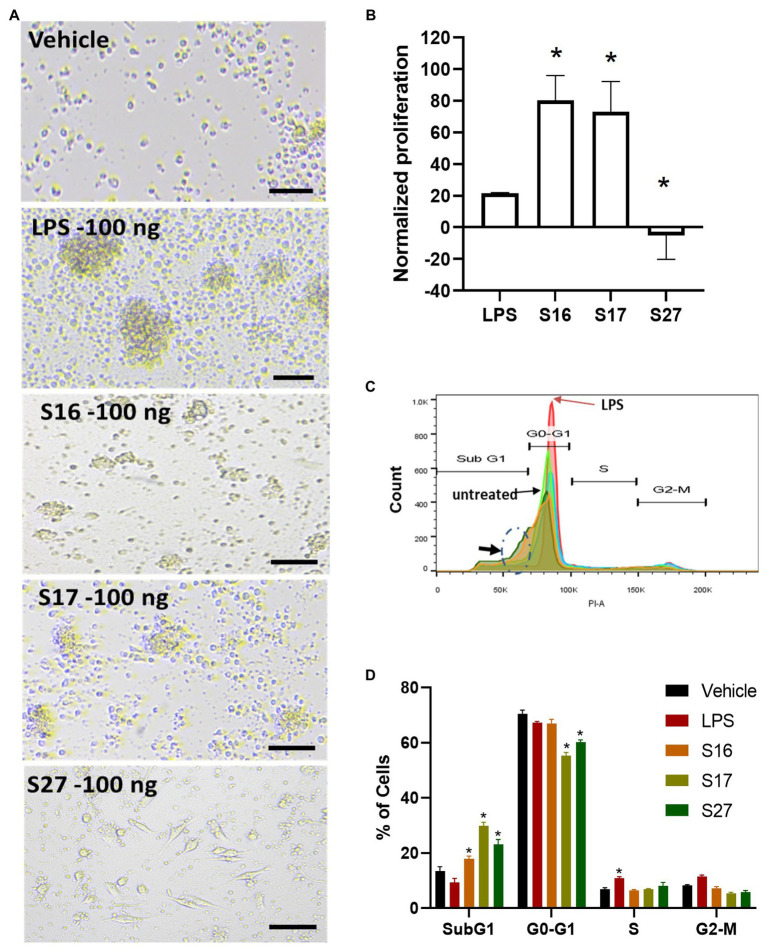
PBMM response to S16, S17, and S27 peptides. **(A)** Changes in the morphology of macrophages in response to peptides. Scale bar = 100 um. **(B)** XTT proliferation assay. The results were analyzed using one-way ANOVA and presented as mean ± SEM. * *p* < 0.05, considered as significantly different from other groups. **(C)** Flow cytometry histogram indicating the shift in cells population from G1 phase to sub G0 phase (dashed circle marked with arrow). **(D)** Cell cycle analysis of macrophages incubated with the peptides. The results were analyzed using two-way ANOVA and presented as means ± SEM of three independent experiments. * *p* < 0.05, considered as significantly different from other groups.

**Figure 6:**
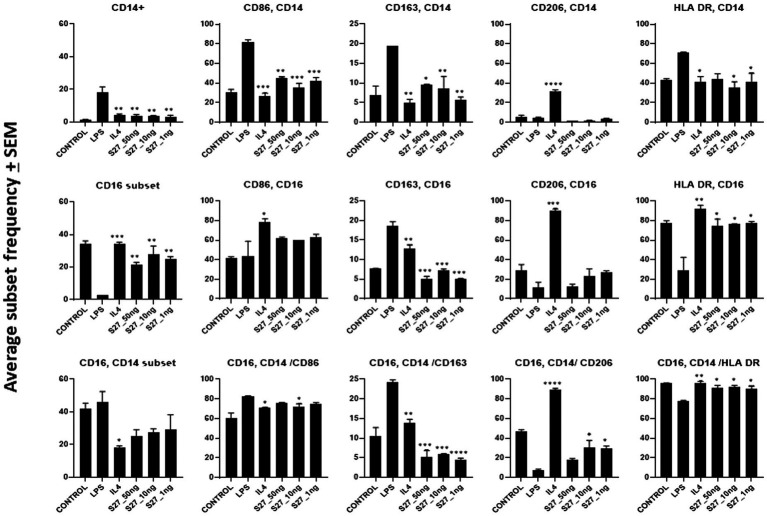
Flow cytometry analysis of PBMM treated with LPS and IL-4 positive controls, and S27 at 1, 10, and 50 ng compared to untreated control. Phenotypic profiling of macrophages following treatment with secreted peptides was examined by assessing the differential expression of key macrophage-related markers including CD14, CD16, CD86, CD163, and CD206. The results were analyzed using one-way ANOVA and presented as mean ± SEM. * *p* < 0.05, ** *p* < 0.01, *** *p* < 0.001, and **** *p* < 0.0001, which is significantly different from another group.

To investigate whether the antigenic peptides S16, S17, and S27 have an immunomodulatory activity, PBMMs were separately treated with these peptides in comparison to LPS, which was used as a positive control. Our data showed that peptides S16 and S17 induced dose-dependent morphological changes and significant proliferation (*p <* 0.01) in PBMMs, which was comparable to that observed with LPS (Figures 5A,B). Such cell appearance is consistent with macrophage polarization to a pro-inflammatory (M1) phenotype (Qi et al., 2016). In contrast, peptide S27 caused attachment and fibrocystic-like morphological changes consistent with the morphology of anti-inflammatory (M2) macrophages (Figures 5A,B; Buchacher et al., 2015). Cell cycle analysis showed a significant (*p <* 0.01) percentage of PBMMs at the sub G0 phase when treated with peptides S17 and S27, possibly indicating terminal differentiation of such macrophages to M1- and M2-like morphology, respectively, (Figures 5B–D). To further confirm the ability of peptide S27 to induce M2 polarization, PBMMs incubated for 8 days in the presence of peptide S27 were phenotypically profiled for the expression of CD14, CD206, CD163, HLA-DR, CD16, and CD86. LPS and IL-4 treatments served as positive controls for M1 and M2 subsets, respectively. Macrophages subpopulations with CD14^+^CD16^−^, CD14^−^CD16^+^, and CD14^+^CD16^+^ phenotypes were investigated to categorize macrophages classical, non-classical, and intermediate subsets, respectively. Each subset was then investigated for their expression of CD86, CD163, CD206, and HLA-DR (Supplementary Figure 2). S27-treated PBMMs demonstrated similar expression profile to IL-4, vis-a-vis CD14, and CD16 expression (Figure 6). The expression of CD86 and HLA-DR were reduced in CD16+ cell subset (Figure 6). Surprisingly, the expression of CD163 and CD206 was downregulated in S27-treated macrophages as opposed to IL-4-treated cells (Figure 6), indicative of specific immunomodulatory effects of the S27 peptide.

### Peptide S27 Induced an M2-Like Polarization by Binding to IL-4/IL-13 Receptor and Activation of STAT-6 Protein

**Figure 7:**
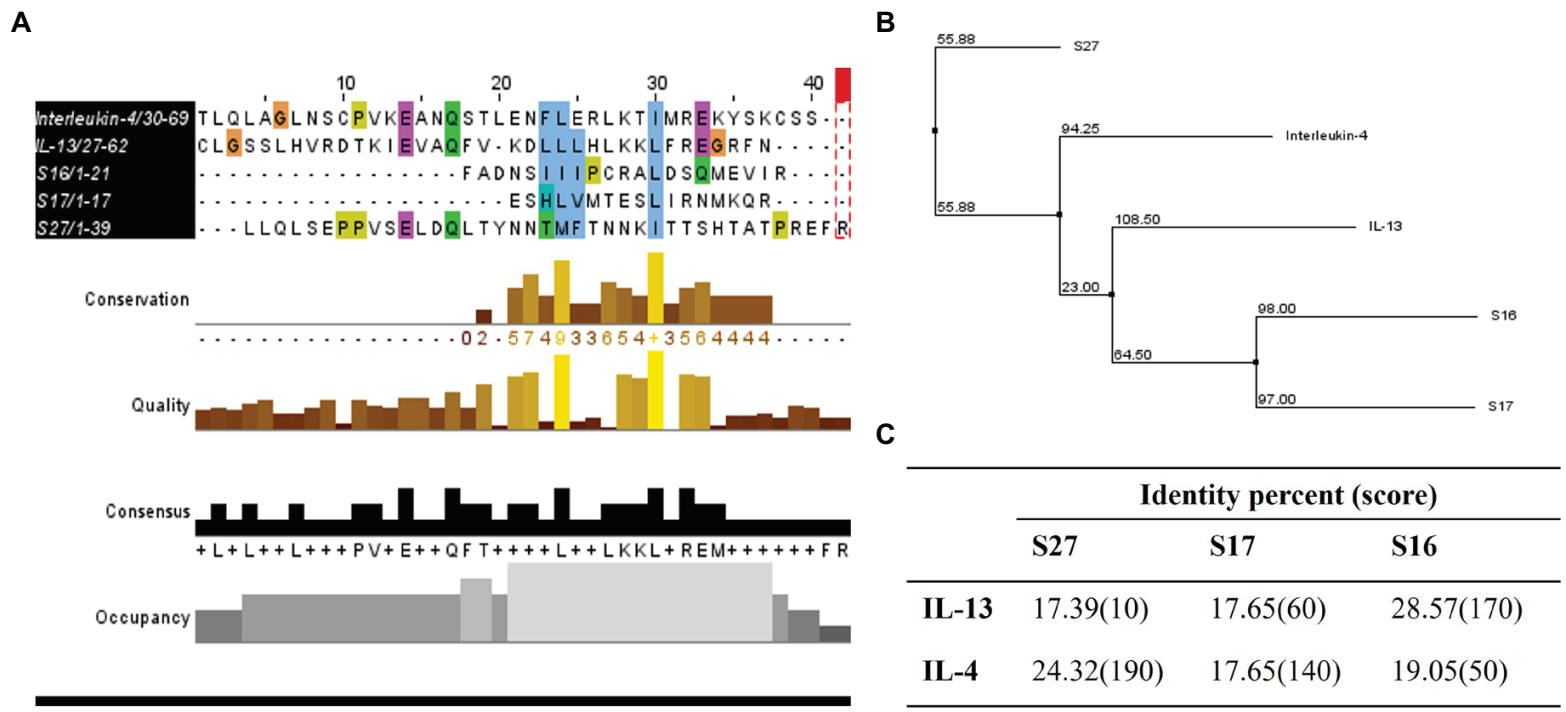
Homology studies between S16, S17, S27, and IL-13 and IL-4. **(A)** Sequence alignment of tested proteins using ClustalOmega and MuscleWS. The colors represent the level of conservation between protein sequences. The blue colored residues are conserved between all proteins (high conservation), while the green and pink are more conserved between S27 and IL-4/IL-13, and the yellow residues are more conserved between S27 and IL-4 (low conservation). Consensus sequence is the most frequent amino acid residues in the alignment, while the occupancy represents the level of the primary consensus binding site motif. **(B)** Neighbor-joining tree using BLOSUM62. **(C)** Pairwise alignment to calculate the identity percentage. Jalview 2.11.1.4 software was used for visualization.

To investigate the immunomodulatory mechanism of *R. arrhizus* var. *delemar* peptide S27 beyond the induction of M2 polarization, we conducted computational homology modeling. The IL-4/IL-13 receptor complex is a major immunomodulator in macrophages which can initiate STAT-6 phosphorylation and hence initiating macrophage differentiation toward M2-like morphology (Martinez and Gordon, 2014; Kim et al., 2018). To test which peptide possesses close similarity to IL-4 or IL-13, ClustalOmega bioinformatics analysis was applied. The results indicated the presence of a shared domain between all tested peptides and IL-4 and IL-13 (Figure 7A), while S27 was more closely related to IL-4 and S16 and S17 were grouped together with a closer relation to IL-13 (Figure 7B). Pairwise alignment revealed that S27 has 24.32 and 17.39 percent identity with IL-4 and IL-13, respectively (Figure 7C). Furthermore, a phosphorylation domain in S27 was predicted at a score level ~ 0.994 using NetPhos – 3.1.

**Figure 8:**
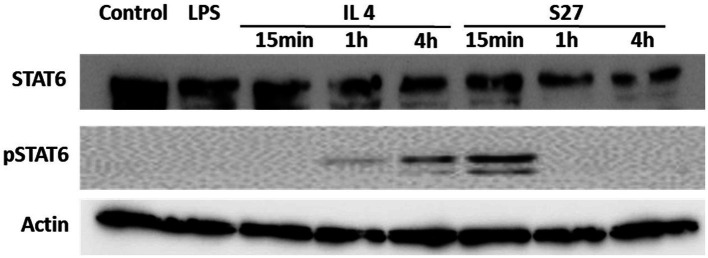
Western blot analysis of the expression of STAT-6 and pSTAT-6 in PBMMs treated with IL-4 or S27. Cell lysates were prepared from PBMMs treated with IL-4 (15 ng/ml) or S27 (100 ng/ml) for 15 min, 1 h and 4 h. Cell lysates of untreated PBMMs or PBMMs treated with LPS (100 ng/ml) for 15 min served as negative controls. B-actin was used as a loading control. Data shown is representative of 3 replicates.

**Figure 9:**
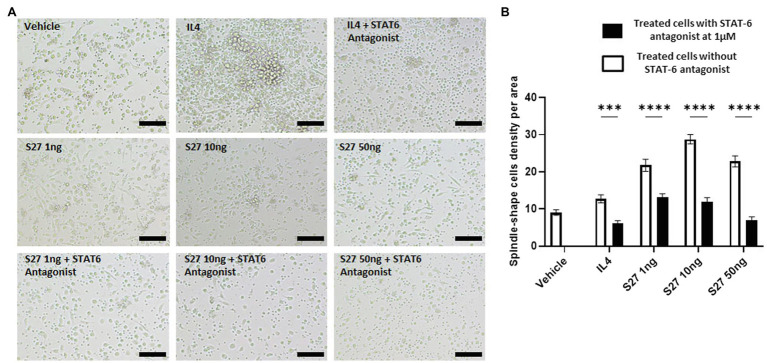
Morphological changes of macrophages in response to different treatments. **(A)** microscopy-based detection of treated macrophages. Cells were treated with different concentrations of S27 (1, 10, and 50 ng/ml), S27 mixed with STAT-6 antagonist. Untreated cells and IL-4 were used as negative and positive controls, respectively. Scale bar = 50 um, magnification 10×, Olympus microscope. **(B)** Counts of macrophages with spindle shape, represented as count per area for more than three fields. White bars representing vehicle, S27 1, 10 and 50 ng. Black bars represent same treatments with the presence of STAT-6 antagonist. The results were analyzed using two-way ANOVA and presented as means ± SEM of three independent experiments. *** *p* < 0.001 and **** *p* < 0.0001, which is significantly different from the other group.

Engagement of the IL-4/IL-13 receptor complex was previously reported to initiate STAT-6 phosphorylation (Kim et al., 2018). Our computational homology data predicted that S27 may have a phosphorylation capacity. In support of these computational predictions, Western blot analysis showed an early STAT-6 phosphorylation in the S27-treated macrophages at 15 min (Figure 8; Supplementary Figure 3). STAT-6 phosphorylation was initiated at 1 h and significantly increased after 4 h in IL-4-treated macrophages. This is concordant with the significant increase in the density of spindle-shaped macrophages seen in the presence of either IL-4 or S27 (Figures 9A,B; Qi et al., 2016). Addition of a STAT-6 inhibitor in the presence of peptide S27 or IL-4 significantly reduced the density of spindle-shaped macrophages, which is indicative of M1-like polarization (Figure 9).

### S27 Modulates Cellular Iron Metabolism in PBMMs

**Figure 10:**
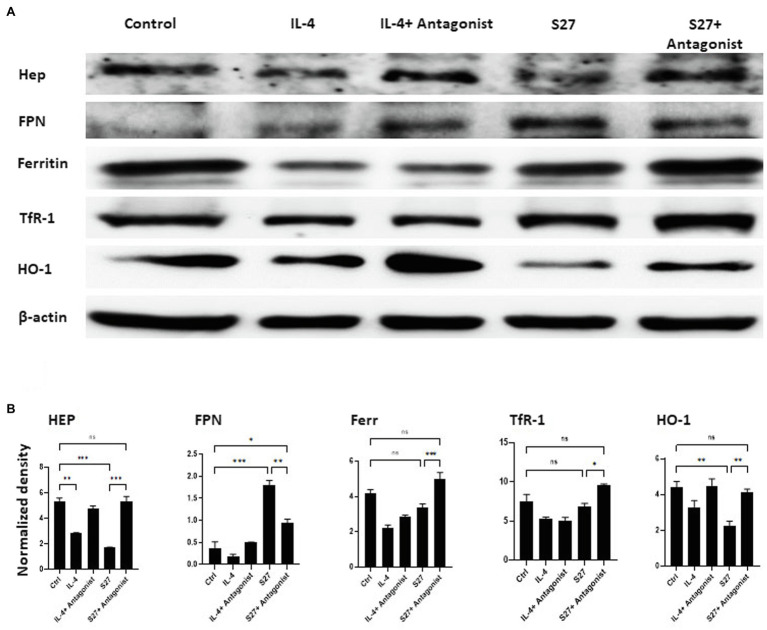
Western blot analysis of the expression of Hep, FPN, Ferritin, TfR-1, and HO-1 in PBMMs cells treated with S27 alone or in the presence of STAT-6 antagonist. **(A)** Cell lysates were prepared from PBMMs treated with IL-4 at 15 ng/ml and in addition to STAT-6 antagonist at 1 μM and S27 at 10 ng/ml/STAT-6 antagonist at 1 μM. As they were cultured for 8 days. The results shown are representative of three experiments. **(B)** The band intensity was measured by densitometry using the Image J software. The results were analyzed using one-way ANOVA and presented as means ± SEM of three independent experiments. *p < 0.05, **p < 0.01 and ***p < 0.001, which is significantly different from other groups.

Macrophages orchestration of iron during *R. arrhizus* var. *delemar* mucormycosis has been shown to play central role in antifungal immunity (Bairwa et al., 2017; Andrianaki et al., 2018). To further investigate the functional response of PBMMs to S27 peptide, the expression of key cellular iron regulatory proteins was profiled (Figures 10A,B). IL-4- and S27-treated macrophages showed a significant reduction in HEP expression. Consistent with downregulation of HEP, S27 significantly upregulated FPN more than 2 folds (*p* = 0.0005), while the treatment with STAT-6 antagonist reduced the upregulation of FPN. Treatment with S27 showed no significant change on Ferr and TfR-1. In contrast, inhibition of STAT-6 upregulated the expression of Ferr and TfR-1 compared to untreated control (*p* = 0.0009 and 0.01, respectively). S27 treatment also resulted in a significant 1-fold reduction in heme oxygenase (HO-1) expression (*p = 0.0027*). HO-1 is the enzyme that catalyzes heme to facilitate the release of heme-bound iron into the cytoplasm. Treatment with STAT-6 antagonist reversed the effect of S27 on HO-1 expression resulting in similar expression to that observed with the control. Taken together, these findings suggest that S27 increases cellular iron release, possibly in support of *R. arrhizus* var. *delemar* growth *in vivo* and hence the progression of infection (Alexander et al., 2006).

## Discussion

Secreted peptides play important roles in fungal pathogenesis. For instance, changes in the fungal secretome, which are host, strain- and environment-dependent, have been implicated in promotion of infection including pathogen survival through nutrient acquisition, virulence, evasion of host immunity, and defense against competing microbes (Suleau et al., 2006).To investigate the secretion of *R. arrhizus* var. *delemar* peptides with possible immunomodulatory effects, the pathogen was grown under two different culture conditions representing two major behavioral phases of fungal pathogenesis, namely, the initiation of infection phase and the hematogenous dissemination-like phase. A hallmark of mucormycosis is the ability of the fungus to cause angioinvasion (Sephton-Clark et al., 2018). Although the pathogenic roles of peptides identified during the initiation of infection are not fully identified, some of them are recognized as nutrient-degrading enzymes (glucoamylase and lipase; Phalip et al., 2005) or as allergens (enolase; Breitenbach et al., 1997). It is generally accepted that microbial pathogens produce and deliver a myriad of effectors to hijack the cellular program of their host (Sanders, 2011) including the secretion of degradable enzymes (Maeda et al., 2005; Mondal et al., 2016). Furthermore, eukaryotic pathogens secrete effectors that modulate innate immunity to favor their survival during infection (Birch et al., 2006; O’Connell and Panstruga, 2006). In this context, screening of peptides from dissemination-like condition using SVMTriP revealed that three 10 amino acids sequences [(PCRALDSQME, from S16), (TESLIRNMKQ, from S17), and (VSELDQLTYN from S27)] were predicted to be antigenic. This suggests that such peptides may contribute to phagosome/inflammasome-mediated degradation, MHCII complex peptide presentation, T helper polarization, B-cell activation and ultimately antibody production, reviewed in (Heung, 2020). Future investigations into these possibilities are warranted.

Infected hosts rely on pro-inflammatory macrophages (M1) as an important line of defense that aims at restricting fungal pathogen dissemination (Andrianaki et al., 2018). Monocyte-derived macrophages residing in tissues are key innate immune cells that phagocytose and digest microorganisms, process and present antigens to T helper cells, and contribute to post-infection tissue remodeling (Gosselin et al., 2014; Xu and Shinohara, 2017). In the present study, we showed that *R. arrhizus* var. *delemar* peptide S27 favors the polarization of anti-inflammatory M2 macrophages by interacting with the IL-4/IL-13R complex and phosphorylating STAT-6. Macrophages have shown wide plasticity in their ability to get activated *via* alternative pathways triggered by the engagement of a whole host of TLRs and/or IL-4/ IL-13 receptors (Martinez et al., 2009). This could differentially lead to CD4+ T helper-dependent pro- or anti-inflammatory responses (Borthwick et al., 2016). Activation of the IL-4/IL-13 pathway was previously shown to play an important role in air-way hyperactivity associated with allergen-induced hypersensitivity (Ramalingam et al., 2008), a condition characteristic of mucormycosis (West et al., 1995). Therefore, secreted peptides, such as S27, may contribute to mucormycosis pathogenesis by dampening the host immune response *via* M2 polarization of macrophages.

Library selective approaches are commonly used to identify peptides with potential activation and functional modulation of macrophages (Cieslewicz et al., 2013). Analysis of peptides with immunomodulatory function from *R. arrhizus* var. *delemar* secretome library is lacking. Further investigation is expected to explain how *R. arrhizus* var. *delemar* can evade mucosal immunity through its immunomodulatory peptides and at the same time can be potentially eliminated once disseminated in immunocompetent hosts. Moreover, understanding the expression patterns of these peptides during the dissemination of mucormycosis may aid in the development of therapeutic approaches that selectively target peptides, which dampen protective pro-inflammatory immune responses.

**Figure 11:**
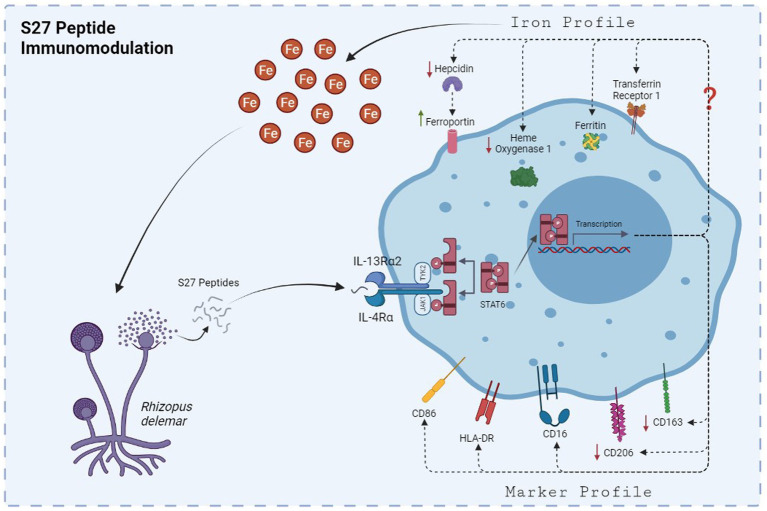
The mechanism by which S27 peptide caused anti-inflammatory modulation to PBMMs. Upon dissemination, secreted S27 interact with IL-4/IL-13R complex causing and early phosphorylation of STAT6. Macrophages will polarize into M2-like phenotype and modulation in iron handling proteins that favors the release of iron into the milieu of mucormycosis that support further persistence and dissemination.

Macrophage polarization is usually indicated by a change in cell morphology (spindle- vs. round-shaped cells) along with functional and phenotypic characteristics as well as distinct secreted cytokines profiles (McWhorter et al., 2013; Toniolo et al., 2015). Previous work has shown that IL-4-dependent initiation of M2 polarization is driven by the JAK1/STAT-6 signaling pathway (He et al., 2020). Our results showed that S27 peptide polarized PBMMs to an M2-like phenotype through early phosphorylation of STAT-6 (Modeled in Figure 11). STAT-6 pathway was previously shown to be essential for M2-like macrophage polarization (Gong et al., 2017; Waqas et al., 2019). Inhibition of STAT-6 pathway activation was also reported to skew macrophage polarization to the pro-inflammatory M1-like phenotype (Kim et al., 2018) and promote phagocytosis (Pereira et al., 2019).

S27 treatment induced a non-classical macrophage phenotypic profile (CD14^low^CD16^high^). Non-classical macrophages possess some of the anti-inflammatory characteristics of M2 cells, including the resolution of inflammation and tissue remolding (Ross et al., 2021) along with their iron release phenotype (Corna et al., 2010). Additionally, HLA-DR and CD86 expression was assessed to ascertain the macrophage subset. The expression of HLA-DR on macrophages indicates an efficient antigen presentation that is accompanied by the upregulation of activation markers, such as CD86, to mediate T helper lymphocyte responses (Eligini et al., 2015). The modulation of these markers in S27-treated PBMMs in comparison to IL-4 treatment further implies immunomodulatory potential toward fungal survival. Phenotypic marker analysis of PBMMs also demonstrated the presence of a distinct marker expression profile (Figure 6). Our findings documented a decrease in CD163 and CD206 expression following S27 treatment. The downregulation of CD163 and CD206 in macrophages with non-classical activation profile is not readily understood. CD163 is a scavenger receptor for haptoglobin/hemoglobin complex, considering its scavenging properties, it is possible that its downregulation by S27 contributes to release of heme-derived iron to support pathogen growth and infection (Ibrahim et al., 2012; Andrianaki et al., 2018). CD206, a mannose C-type lectin receptor on macrophages and other innate immune cells, has been amply studied and is known to play an important role in fungal pathogen binding and internalization into macrophages (De Ruiter et al., 1994; Allavena et al., 2004; Azad et al., 2014). Therefore, S27-mediated downregulation of CD206 may be viewed as an *R. arrhizus* var. *delemar*-dependent mechanism that promotes evasion of host defenses and persistence. Hypoxia was previously shown to enhance mucormycosis (Chung and Lee, 2020) and was recently highlighted as a key factor in lethal mucormycosis in severe cases of SARS-CoV-2 patients. Hypoxia was also reported to downregulate CD206 surface expression on macrophages and dendritic cells resulting in reduced dextran internalization potential (Raggi et al., 2017). Taken together, these observations appear to further substantiate the possibility that S27 subdues the antifungal immune response of the host.

It appears that the effects of fungal S27 to modulate the phenotypic profile of macrophages is dependent upon, or perhaps a consequence of, its ability to alter the cellular iron status in macrophages. Profiling of proteins regulating iron in macrophages treated with S27 showed reduced hepcidin and increased FPN expression. These changes signify the release of iron (Ibrahim et al., 2007), which is likely to enhance pathogen virulence and/or M2 macrophages (Corna et al., 2010). In contrast, treatment of macrophages with STAT-6 inhibitors antagonized the effect of S27, leading to M1-like pro-inflammatory phenotypic characteristics that associate with iron sequestration and fungicidal activity with increased levels of hepcidin, downregulated FPN along with increased Ferr, TfR-1, and HO-1 expression (Figures 10, 11; Sica and Mantovani, 2012). It is well established that hepcidin is an acute phase inflammatory protein that upregulates in inflamed macrophages in response to IL-6 or LPS treatments (Peyssonnaux et al., 2006; Theurl et al., 2008). Downregulation of FPN, HO-1 expression, and increased ferritin levels occurs in M1 macrophages (Corna et al., 2010). Overall, this is in line with our previous work which has shown that macrophage iron sequestration upregulates host immunity to prevent *Rhizopus* growth within the macrophage (Andrianaki et al., 2018).

## Conclusion

In summary, consistent with the requirements to establish an infection, the secretome of *R. arrhizus* var. *delemar* interacting with alveolar epithelial cells contained several predicted lytic enzymes. In contrast, more toxin-like peptides with immunomodulatory effects dominated the secretome of *R. arrhizus* var. *delemar* when grown under limited oxygen supply to mimic hematogenous dissemination. Particularly, the S27 peptide was found to disrupt the anti−/pro-inflammatory balance in macrophage subsets and induce macrophage polarization toward an M2 phenotype that typically promotes fungal infection. Switching of iron metabolism to increase cellular iron availability as revealed by our study is consistent with the need of fungal spores to survive the phagolysosomal environment and kill macrophages. Collectively, these results highlight the importance of S27 secreted peptide in *Rhizopus* virulence and may represent a promising therapeutic target in mucormycosis.

## Data Availability Statement

The original contributions presented in the study are included in the article/**Supplementary Material**, further inquiries can be directed to the corresponding authors.

## Author Contributions

SS and MM conceived and planed the experiments to investigate the original idea. EE-L developed the computational modeling studies. AH helped in running the experiments. BF, AA-Q, AA-R, and SD conducted the flow cytometry, western blotting analysis, and PBMM analyses. A-NE-S, MH, and AI helped in planning the project. MM ran the cell cycle, PBMM phenotypic, and morphological analysis. AI provided funds and critical materials. SS, EE-L, AA-Q, BF, AH, AA-R, SD, A-NE-S, MH, AI, and MM helped in data analysis and interpretation and wrote and revised the manuscript. All authors contributed to the article and approved the submitted version.

## Funding

This work was supported by grants 2101110147, 1801050130, and 1801050232 to SS and MM from the University of Sharjah, Sharjah, UAE, and by a Public Health Service grant R01AI063503 to AI.

## Conflict of Interest

AI owns shares in Vitalex Biosciences, a start-up company that is developing immunotherapies and diagnostics for mucormycosis.

The remaining authors declare that the research was conducted in the absence of any commercial or financial relationships that could be construed as a potential conflict of interest.

## Publisher’s Note

All claims expressed in this article are solely those of the authors and do not necessarily represent those of their affiliated organizations, or those of the publisher, the editors and the reviewers. Any product that may be evaluated in this article, or claim that may be made by its manufacturer, is not guaranteed or endorsed by the publisher.
