# Supplementary material for: Novel Secreted Peptides From Rhizopus arrhizus var. delemar With Immunomodulatory Effects That Enhance Fungal Pathogenesis
Source: Front Microbiol. 2022 Mar 21;13:863133. doi: 10.3389/fmicb.2022.863133 (PMC8977774; doi:10.3389/fmicb.2022.863133)
Supplement: Supplementary file 1 [file Data_Sheet_1.pdf]

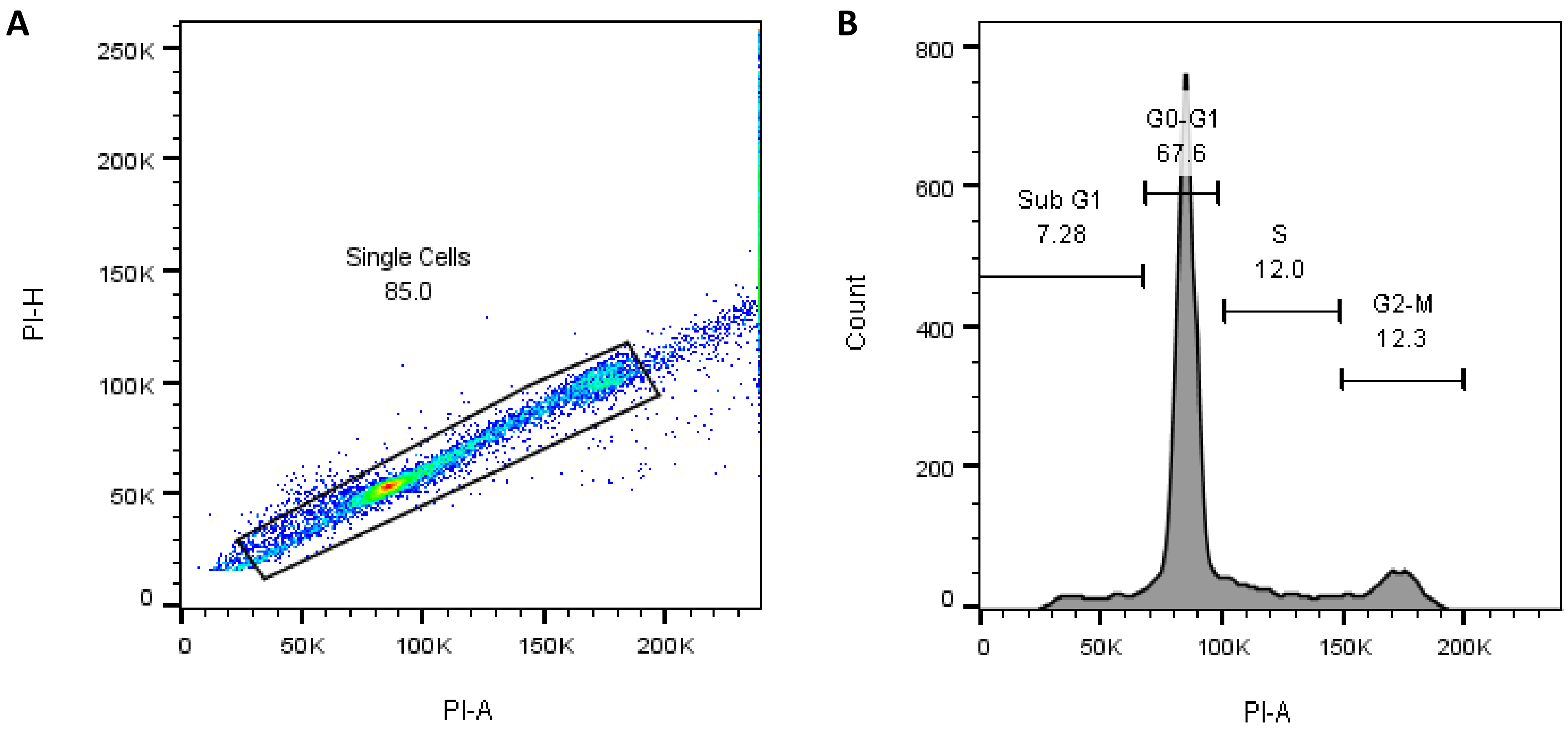

Supplementary Figure 1. Gating strategy used for cell cycle analysis. Representative plots are shown. (**A**) Single cells were gated using Propidium Iodide – hight (PI-H) versus PI-area (PI-A). (**B**) Then different cell counts were determined based on their PI-A fluorescence intensities.

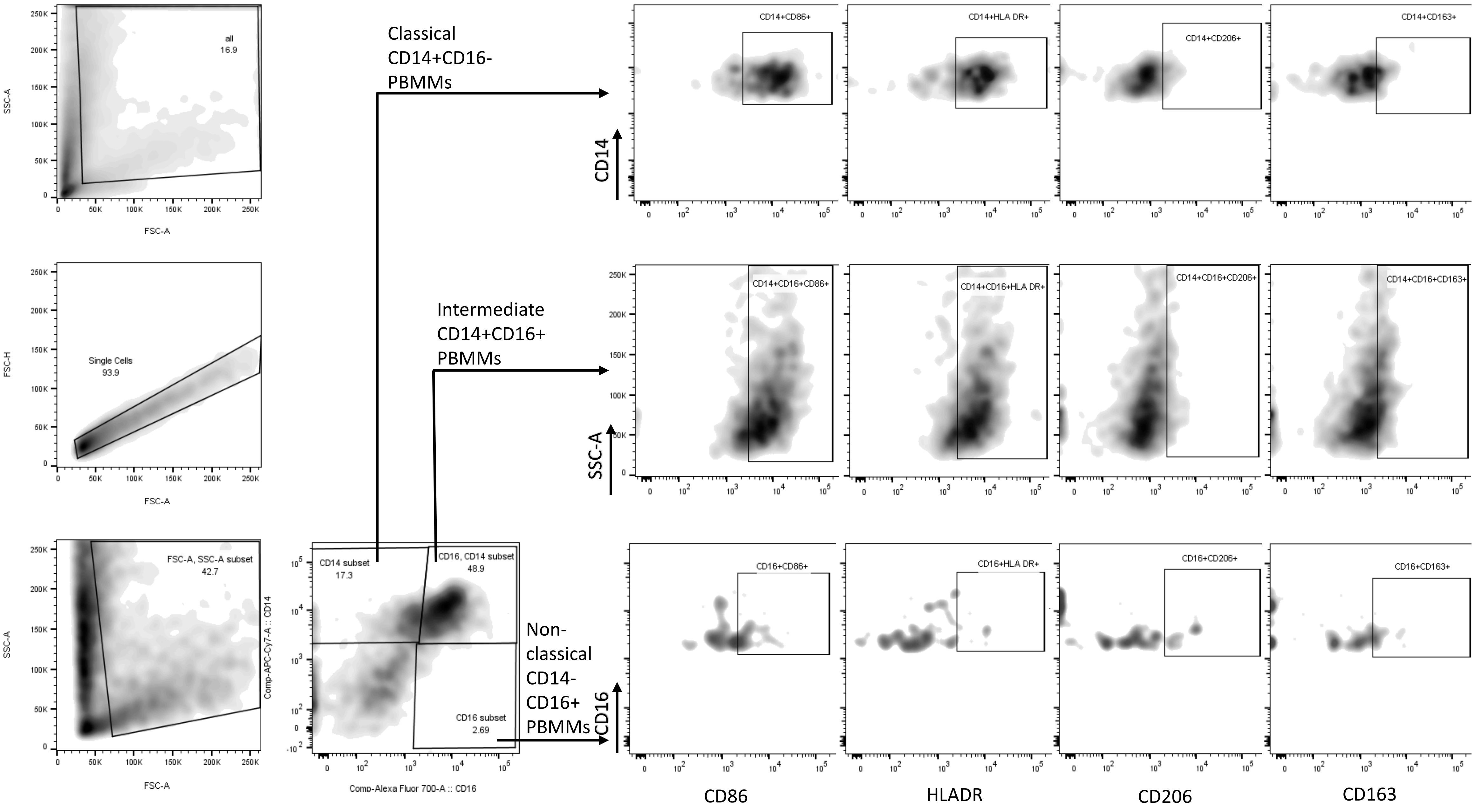

Supplementary Figure 2. Gating strategy used for PBMMs phenotyping. Representative plots are shown. Single cells were gated followed by determination of CD14 and CD16 subsets. Each subset then investigated for the expression of CD86, HLADR, CD206 and CD163. Side scatter-A (SSA-A) was used to discriminate phenotypes of intermediate macrophages (CD14+CD16+).

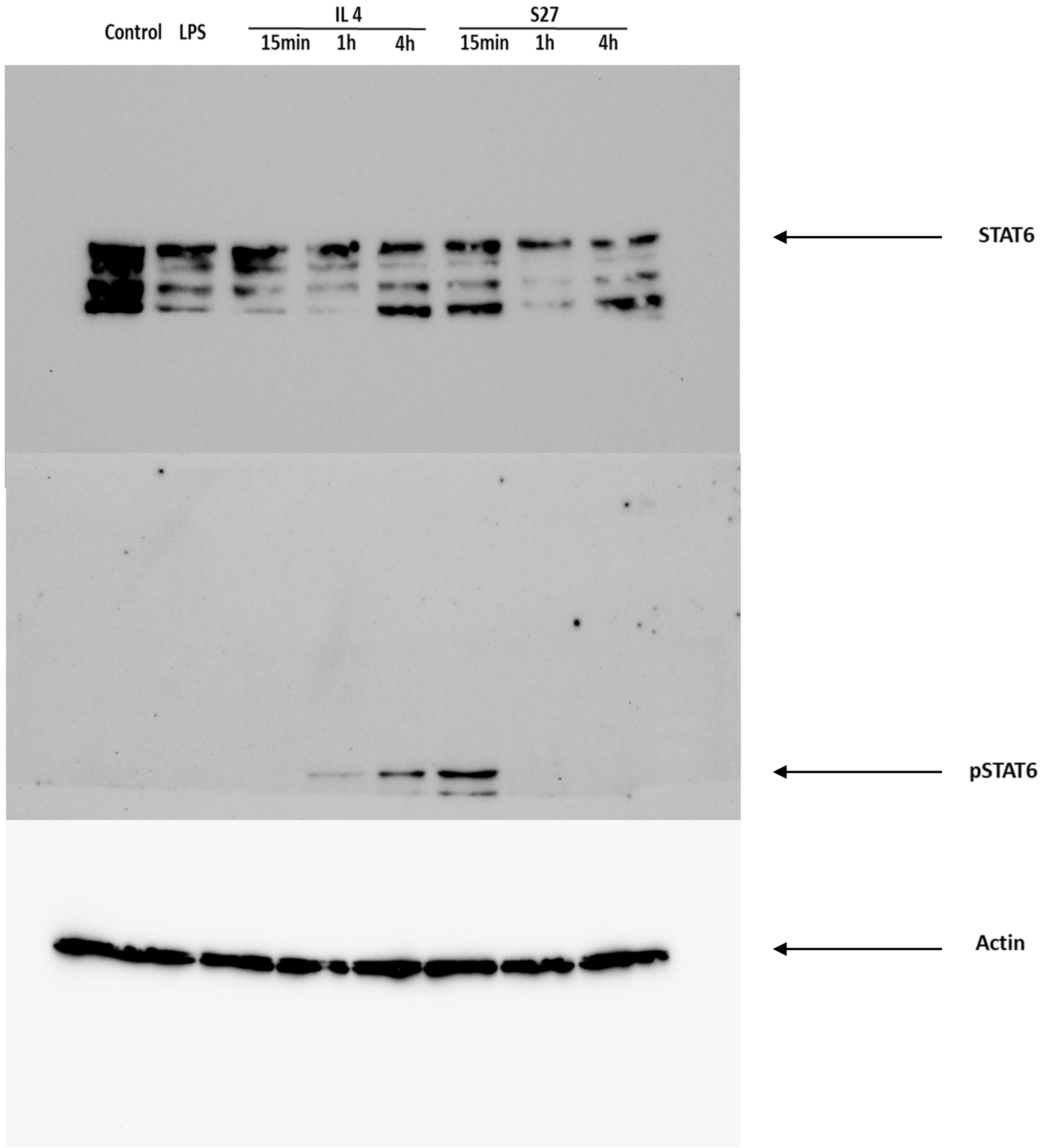

Supplementary Figure 3. Western blot analysis of the expression of STAT-6 and pSTAT-6 in PBMMs treated with IL-4 or S27.
